# The Following Comments Were Elicited by the Perusal of an Essay, Read in Part, by Dr. C. A. Harris, of Baltimore, before the American Society of Dental Surgeons, at Their Annual Meeting, Held in Boston, July 20th, 1842

**Published:** 1843-06

**Authors:** H. H. Hayden

**Affiliations:** Dental Surgeon, of Baltimore.


### ARTICLE V.

*The following comments were elicited by the perusal of an Essay, read in part, by Dr. C. A. Harris, of Baltimore, before the American Society of Dental Surgeons, at their annual meeting, held in Boston, July 20th, 1842.* By H. H. Hayden, M. D. Dental Surgeon, of Baltimore.

(Concluded from page 226.)

In the next place, not wishing to be chargeable with prolixity, or a propensity to indulge in useless animadversions, we shall leave a number of passages that really invite some passing remarks, and limit our attention to the following subject, viz. "Of inflammation of its lining membrane."—Page 32.

Upon this head the doctor observes,—1st. "The most simple form of disease that occurs in this cavity is inflammation of its lining membrane, and this, in most instances, may be said to precede all others."—"It often subsides spontaneously, but when it continues for a long time it is apt to become chronic, and then it not unfrequently gives rise to other and more formidable kinds of disease."—"When unattended by any other marked affection, either local or constitutional, it is easily cured."—Page 25.

2d. "Inflammation, when not complicated with any other morbid affection, is the most simple form of dissease to which the pituitary membrane of the antrum maxillare is subject. It precedes and accompanies all others that are here met with, and it will therefore be proper to offer a few remarks upon it before entering upon the consideration of those of a more aggravated nature."—Page 32.

3d. "Shielded as the pituitary membrane of this cavity is from most of the acrid and irritating agents to which it is exposed in the *nasal fossa* and some of the other cavities of the body, it would rarely here be affected with inflammation, were it not that it is frequently acted upon by other *morbid excitants,* whose immediate influence seldom extends beyond the alveolar border and face; but, exposed as it is to the action of irritants of a *very aggravating kind,* inflammation of it is of frequent occurrence, more so, perhaps, than many are aware of, and it is to this that those deep-seated pains in the superior maxilla and face, which are usually denominated rheumatic, are, in many instances, attributable."—Page 32.

Respecting the first proposition, viz. "The most simple form of disease that occurs in this cavity is inflammation of its lining membrane," we could have wished that our friend, the doctor, should have given us at least some idea of the cause, nature and degree of inflammation to which the tissues of the maxillary sinus are liable, and which, "in most instances, may be said to precede all others." Now we can easily imagine and safely say that if the tissues of one or both of the sinuses *have been* and are still in a perfectly normal state, and inflammation from any cause is lighted up, it may truly be said to "precede all others," as disease has not before existed in those cavities; but this account affords us no satisfactory information respecting the nature and cause of the existing inflammation in those tissues. Indeed, it seems necessary, in order to determine these two points to ascertain what inflammation is, or in other words what constitutes inflammation. The causes are extremely various, but not so difficult of solution; and the consequences and degrees of extent to which parts subject to its influence are liable, we are already but too well acquainted with. It then remains to determine, as well as the case will admit, what inflammation is, in order to determine the correctness of the doctor's views on the abnormal condition of the tissues of the maxillary sinuses.

Dr. Thompson, whose opinions and celebrity seem to stand highest in this department of medical science, says: "In the *incipient* stages of inflammation, it obviously depends on the influx of an unusual quantity of blood into the vessels of the part *becoming inflamed;* the redness may be produced either by the distension of those minute vessels which naturally convey a small quantity of red blood, or by the particles entering into vessels which in their healthy state *admit only serum.*"—Page 41. Again,—"The enlargement in the capacity of smaller blood vessels, during the state of inflammation, and the increase in the number of those which become capable of conveying red blood, are facts which have been long known to pathologists."—Page 42.

These are considered as amongst the most prominent or leading phenomena of incipient inflammation. Let us now see how far our friend's, the doctor, views will quadrate with Dr. Thompson's. Unfortunately, our friend, the doctor, in the first proposition, does not assign any cause of inflammation, merely observing that "the most simple form of disease that occurs in this cavity is inflammation of its lining membrane." Moreover,—"It often subsides spontaneously, but when it continues for a long time it is apt to become chronic, and then it not unfrequently gives rise to other and *more formidable* kinds of disease."

So far as respects the "most simple form of disease that occurs in this cavity," viz. "inflammation of its lining membrane," there is no material difference of opinion, since it, "in most instances, may be said to precede all others," therefore in its nature primary, and differing in no degree from Dr. Thompson's description of incipient inflammation; and as to the *cause,* it does not require a great depth of research or power of intellect to satisfy the inquirer after truth, that the causes of inflammation, in this as well as in many other cases, are not only numerous but extremely varied; such as, amongst others that have been enumerated by different writers, blows, falls, or violence inflicted upon these parts, diseases seated upon and around the roots of the teeth, thereby extending to, and affecting consecutively the tissues or lining membranes of the maxillary sinuses, violent colds that are seated upon, and involve the mucous membranes and other tissues of not only the maxillary, frontal and other sinuses, but of every cavity of the head, and also of other parts of the body. The cause or causes and nature of simple inflammation in the tissues of those cavities being known, and we believe understood, the therapeutical indications are rendered plain and easy; but not so with the facts accompanying or involved in the phenomena mentioned in the third proposition, and also in the remarks which follow, viz. "Exposed as it (the maxillay sinus) is to the action of irritants of a *very aggravated* kind, inflammation of it is of *frequent* occurrence, more so perhaps than many are aware of," &c. (Journal of Dental Science, vol. 3, p. 32.)

Amongst these the doctor has enumerated "the acrid and irritating agents to which it (the maxillary sinus) is exposed, in the nasal fossa and some of the other cavities of the body," as febrile and gastric affections, eruptive diseases, such as measles, small-pox, &c. &c. "syphilis, an excessive and protracted use of mercurial medicines, a scorbutic or scrofulous diathesis of the general system." From these premises a question presents itself for consideration, and the result of which, we hope, will enable us to determine the nature and cause of complicated diseases of the maxillary sinuses, and the extent to which they are to be admitted. In the first place, we wish to be informed respecting the "acrid and irritating agents to which it (the pituitary membrane) is exposed in the nasal fossa;" and, moreover, respecting "the action of irritants of a *very aggravating kind,*" by which "inflammation of it (the tissues of the sinuses) is a frequent occurrence."

We are well aware that a violent cold, as we have already remarked, will occasion a derangement in the economy of *all the cavities* of the head, accompanied with a copious discharge of secretions from the nose and eyes, and, in some instances, so acrid as to excoriate the skin, in its exit from the nose and from the eyes; but this, though often distressing and painful, we consider, in its earlier stages, as the most common and "simplest form of disease that occurs in this cavity, (the maxillary sinus,)" and is doubtless idiopathic, and, as my friend, the doctor, remarks, "it often subsides spontaneously," and more especially so, even if both sinuses are involved, with seasonable and judicious nursing. But whence come the "other morbid excitants," or the "irritants of a very aggravating kind," by which "inflammation of it (the tissue of the sinuses) is of frequent occurrence," and which, we suppose, constitutes complicated disease of the antrum." Of these, however, we are not informed, nor can we come to any definite conclusion respecting them, unless they be a consequence of "other *marked affections,* either local or constitutional," such as "febrile and gastric affections, eruptive diseases, as measles, small-pox, &c. &c. syphilis, and excessive and protracted use of mercurial medicines, a scorbutic and scrofulous diathesis of the general system." With regard to the effect or agency of scrofula in causing exostosis, &c. of the bones, the doctor quotes Sir Astley Cooper as giving a different opinion, viz. "Sir Astley Cooper declares that no evidence has yet been adduced to prove that the former (scrofula) is ever concerned in its production," viz. exostosis, &c. (Journal of Dental Science, vol. 3, p. 179.)

Now as "febrile and gastric affections," if exercising any influence at all in lighting up disease in *any* of the cavities in the system, we may reasonably conclude that it would be general; but disease of the frontal sinuses, of the mucous tissues of the nares and of the mouth, would be alike inflamed by the state and condition of the general economy. We ask, is it so, or is this the fact? We answer, according to our belief and long experience, No! But should disease of the simplest form be lighted up, in one or both of the maxillary sinuses, when the general system is otherwise in perfect health, should disease of a gastric or febrile character supervene in this state, we have no doubt that the disease, from simple inflammation, would be much retarded in any attempts for its restoration to a healthy condition.

In the next place we will offer a few remarks upon the subject of eruptive diseases, such as measles, small-pox, varioloid, and others of a similar character. We believe that during the prevalence of either of these affections, no surface of the human body is exempt from its influence; that it is made manifest at and upon every accessible point, from the crown of the head to the thickest part or portion of the soles of the feet, even under the toe and finger nails, where it, the pustule of varioloid or small-pox, remains until cast off by the growth or renewal of those appendages, viz. the toe and finger nails.

Having witnessed these facts, we cannot for a moment withhold our assent to the opinions of Tenon, Bunon, and Bourdet, who, in the great hospitals of Paris, examined the effects of those and all other diseases upon the teeth, before and after their appearance, and of the maxillary sinuses with the most scrupulous attention. Of these facts then we can entertain no doubt. But these diseases being of an evanescent character, and prevailing in the mouth, nares-maxillary, or frontal sinuses, soon pass away, and leave the parts in, comparatively, a normal state. Nor are we disposed to believe in any other result, except where a pre-existing disease in one of these parts was already established. In such a case the latter in consequence have been more or less aggravated, in which state, it may be justly termed a complicated disease, though in general located in and confined to one of the maxillary sinuses.

The next subject that presents itself for our consideration, as being either a remote or proximate cause of diseases of the maxillary sinuses, is that of syphilis, the most odious and objectionable term that can be selected, as applicable in any degree as an accessory. Indeed, the very use of the term, in this case, admits the inference that whoever is subject to a disease of the maxillary sinus, whether a male or female of the most chaste and unblemished character in society, is chargeable with palpable indiscretions; therefore not deserving the commisseration of any one.

Let us, for a moment examine the subject in order to ascertain the progress, and effects of syphilis upon the general system or parts thereof, most liable to its injurious effects. In attempting this, we shall not engage in a critical investigation of the various features which this disease assumes—suffice it to say, that unless seasonably checked, the whole circulating system becomes saturated with its contaminating influence, and which rapidly tends to a general dissolution.

But during the prevalence of its aggravated symptoms, we seldom find the maxillary sinuses, either primarily or secondarily the seat of its attacks, but rather, if at all, the soft and delicate parts of the throat, and posterior parts of the mouth, as the velum palati, the uvula, and added to which are blotches of the delicate and unexposed parts of the skin about the head and under the hair. If mercury is administered to check its progress, or subdue its desolating influence, we soon witness its attacks upon the *delicate spongy bones of the nose—the roof of the mouth*, &c. but very seldom, even in the most desperate cases do we witness or hear of an attack upon either of the maxillary sinuses. Moreover, we think it highly improbable that either of those cavities was ever primarily affected by this disease, or if at all, only secondarily; in which case it should, by no means be considered as pertaining to a syphilitic disease but, on the contrary, as the doctor himself observes, rather as a consequence of, "or unhealthy predisposition of the body."

As it respects the "excessive and protracted use of mercurial medicines," being, in any degree accessory to the several complaints incident to the maxillary sinuses, we are by *no means* willing to admit, to the extent to which many are disposed to believe, as a proximate cause of the several diseases incident to those cavities. If at any time, a predisposition to, or in fact an actual disease does exist in one or both of the maxillary sinuses, a long continued use of mercury may tend to aggravate, and thereby occasion what may be termed a complicated form of the malady. Moreover, it is very doubtful whether "a scorbutic or scrofulous diathesis of the general system, ever did disturb the economy of these cavities so far as to occasion, primarily, an acute or chronic disease in those tissues. We are aware that it is very common for persons who write upon the diseases of the mouth, to include amongst them that of scurvy—by some "scorbutus." But it is very rarely that we have witnessed the marked symptoms of real scurvy in the mouth of persons who had enjoyed for any length of time, the blessings of pure water—wholesome food—and an equally free and wholesome atmosphere. It is true, we sometimes witness cases that bear a strong resemblance to a scorbutic diathesis; but the latter is, almost uniformly, a consequence of local irritation, and may be termed the scurvy of neglect on the part of the patient, in as much as one side of the mouth is in perfect health, while the other is diseased and flabby. The former is, in its nature, of a general character, in which both the solids and fluids of the system, are from various causes, in an abnormal condition.

In this state, why is either one or both of the maxillary sinuses any more liable to be affected than the adjacent or surrounding parts, as the mouth, the fauces, the nares, the amygdal glands, or the frontal sinuses, as each and all are lined, or covered with the same tissues, and liable to the very same diseases. This is seldom if ever the case, except from local irritation, and then but temporarily. But few amongst many writers have manifested a better or more familiar acquaintance with real scurvy than Thos. Trotter, M. D. of the royal navy. "The debility of scurvy," says the doctor, "is of so *singular a nature, that nothing seems analogous* to it; certain it is, that no disease is *related to it,* by any *concourse of symptoms or method of cure.* Those medicines which invigorate the system in other cases of debility, have little *effect here.* Wine, the *sovereign* remedy of typhus fevers, gives a momentary stimulus, but *no permanent* relief. It does not *retard* the disease; and it is not even when the mind is *sinking* under *despondency,* desired by the scorbutic patient."—Trotter on

Scurvy, p. 106. And yet this odious and much dreaded disease, together with syphilis, requires excessive and protracted use of mercurial medicines. A scorbutic or scrofulous diathesis, together with protracted inflammatory and bilious fevers, and even dyspepsia, &c. are lugged in to swell the list, and increase the number of evils that are supposed to operate as auxiliaries in promoting the diseases of the maxillary sinuses.

From this view of the subject we find it difficult to explain, and no less so to comprehend the motives which prompted our friend, the doctor, to summon such an array of imaginary causes of the diseases incident to the maxillary and other sinuses of the human head, unless it was to endeavour to mystify the subject as much as possible, and make it appear that not until recently, and that, by a chain of fortuitous circumstances, has the direct rays of the true light of correct pathology and physiology ever been permitted to shine into the dark recesses of these depositories of morbid accumulations of festering tumours and lurking fungi, and made known to the world. 'Tis passing strange indeed.

Of the numerous and varied cases of diseases of the maxillary sinuses that have been reported, and which stand registered upon the pages of medical history, by surgical writers of known celebrity and skill, how many may we reasonably suppose ever gave themselves the trouble to inquire into the real cause or causes of the prevailing disease, or, in fact, to institute a critical examination of the form and structure of these cavities, with similar views? We answer that the number must be small indeed; though if it be true, as our friend, the doctor has said, that "it will be unnecessary to give a full description of this cavity, inasmuch as that has been done by nearly every writer on general anatomy since the middle of the seventeenth century."—Journal, p. 21. Moreover, of the cases so reported, how many may we reasonably suppose were the result of violence inflicted upon or about the parts, as blows, falls, or contusions from some cause or other, which though the source of little uneasiness at first, ultimately terminated in disease of a serious character? In the next place, how many cases may we suppose were the result of an abnormal condition of the mucous and other tissues of one of those cavities, for it must be remarked, that it is a rare occurrence that more than one of them is diseased at the same time? This latter case we consider as, by far, the most fruitful source of the various diseases of these sinuses, and of this we shall have some explanatory remarks to offer.

Now if we admit that the diseases from the first cause, viz. blows, falls, &c. may amount to two-eighths, which we think is ample; and those arising from an abnormal condition arising mostly from colds, diseased teeth, &c. &c. as we shall endeavour to show, amounts to five-eighths; which supposition is by no means improbable or unreasonable, we shall have but one-eighth as the result of constitutional debility, irritability, predisposition, excitability, sympathy, mercury, scrofula, febrile and gastric affections, eruptive diseases, dyspepsia, &c. to make up the cases of complicated "maladies of the maxillary sinuses." This is a small number indeed to make such an astounding report. Having duly weighed, we think, the material points involved in this subject, we will proceed to offer a few explanatory remarks, with a view to show the reasons for a want of conformity with the opinions advanced by our friend, the doctor.

In the first place, we readily accede to the long and well known facts stated; viz. that the most formidable diseases of the maxillary sinuses have evidently been occasioned by violence, such as from blows, falls, contusions, &c. of some kind, or in some way or other. The consequences of which have generally been of two principal, and at the same time different characters. The first is such as have been occasioned by a disturbance or injury to the mouth from fracture, &c. to the periosteum of the diseased sinus, from which we have *morbid growths of hard fibrous tumors*, which if not seasonably arrested or removed, terminate in formidable *bony excrescences*, or *osteo sarcoma*. Such in an especial manner as the case of Moses Lee, as operated upon and reported by Dr. Charles Bell Gibson, see Journal of Dental Science, No. 2, page 138—and the American Jour. of Med. Sciences, p. 277.

The second is such as may result from the same force or accident operating upon the mucous tissues of the same sinus, the result of which would probably be of a vascular, fungoid or humoral character, &c.

As it is not our intention, nor has it been from the first, to offer remarks upon the general views of our friend, the doctor, although there is no want of material; we shall, for the present, dispense with any further reflections upon the two last points.

We proceed then to examine the "most simple form of disease that occurs in this cavity, viz. inflammation of its lining membrane. It precedes and accompanies, says the doctor, (as in the second proposition) all other (viz.) diseases that are here met with." This we presume, will be admitted upon all hands, for we can scarcely form correct views of disease, (except it results from a low state of debility or atrophy) but such as is *preceded* by, and *accompanied* with inflammation. But our business, in the present instance is to examine and inquire into the probable cause of the inflammation in the first place; and secondly, the nature of the parts in which inflammation is seated. Having satisfied ourselves on these points, we shall be better able to form correct views of the variously modified diseases incident to the maxillary sinuses, as resulting from *simple inflammation.* To this state and condition, all the tissues of the cavities of the head, as has been already observed, are liable from various causes; the most common and efficient one is that growing out of sudden changes of temperature. In this state, there is *visible increased vascularity* and *susceptibility* in the membranes of the *nose*, the *fauces*, and the *mouth*, which are perceptible; and to the *sinuses* of the *head and face.* The effect of which is the immediate *closing up of the orifices*, or little *lacunæ* of the mucous glands, which are *clustered* beneath the surface of the buccal membrane, so that no part or portion of the mucus is *permitted* to be *discharged* upon the *surface;* not very dissimilar to the closing up of the pores of the skin in high inflammatory fever. In this state the functions of the mucous glands are continuous; their fluid contents are constantly elaborated and increased in quantity, and, by reason of their retention, their morbid condition also. At length the acrid qualities which they assume form a passage through the orifices of these glands, and are discharged upon the surface, and such is the acrid or corrosive state of the mucus so discharged, that it occasions a white spot in the cuticle and cutis vera, at the *orifice* of the *gland*, similar to that produced by lunar caustic and which denotes the death of the part, and the commencement of *incipient ulceration.* Of this there is no mistake.

It may be observed in any portion of the mucous tissue—but in a striking and singular manner in the buccal membrane, and in the tonsils. In this state the vascularity, and thickening of the mucous tissues of the maxillary and other sinuses are gradually increased, ulceration extended, unless seasonably arrested, and the mucous secretions are poured out in abundance to assist and accelerate the work of disease.

The facts thus stated may be considered as the precursors of almost all the incidents of a morbid character that subsequently the mucous membrane increases, the nasal passage is not only occur in these cavities. As the inflammation and thickening of liable to be, but frequently is closed up, but *never*, we believe, *obliterated*, as both the doctor and Mr. T. Bell assert.* As soon should we look for an obliteration of the mouth or any other important *natural* and *necessary* opening of the body. A temporary obstruction or closing up of an opening or passage into a cavity is not unusual; but an obliteration of a material and necessary opening we consider of a different character. The natural passage being thus obstructed, the mucous secretions, at first limpid, gradually become vitiated, greatly increased in quantity, and of a somewhat purulent character. In this state, the natural tendency of the secretions is downwards, or towards the bottom and lateral cavities of the maxillary sinuses. In this state, subject to the natural heat of the body, they do not remain long before they assume a purulent, congested form, or consistence resembling cheese curd, and in proportion to the time of their retention, they become highly morbid, as well as extremely fetid, qualities which are the unavoidable result of two causes. The first is the natural passage by the nose being partially obstructed, the more fluid parts of the secretions, not having a free exit, are taken up by the absorbents, while the more purulent and congested portion settles down and becomes more firmly impacted in the deep fossæ in the bottom of the antrum. The second is, that the purulent matter, thus congested and impacted in the sinuses of the antrum, becomes daily more and more deleterious and offensive in its condition, and are, at last, capable of pro-

* See T. Bell on the Anatomy, Physiology, &c. p. 253; and Journal of Dental Science, vol. 3, p. 33.

ducing the worst of consequences to the surrounding parts, such, if neglected, as ulceration and destruction of the mucous tissue under and about the vitiated and congested matter, caries, and sometimes displacement of the walls of the antrum, at others, if primarily the result of simple inflammation, tumours variously modified, and sometimes of a painful and threatening aspect. Of these, as before observed, it is not our intention to offer any comments.

In this stage of the business, we consider the case as coming under the legitimate character, (as generally understood,) of confirmed abscess of the antrum. The predominant symptoms of which are, in common, a kind of infiltrating, or discharge of a glairy or muco-purulent fluid from the affected side, occasionally accompanied with a somewhat tenacious and extremely offensive mucus. The secretions, in this case, we consider as differing in no essential point from the secretions of the mucous tissues of any part, when subject to a slight inflammation from a cold. Instance those of the mucous glands of the trachea, the bronchial cells, &c. &c. which, when copious, soon become a congested mass of extremely tenacious or adhesive yellow mucus, which occasions irritation and much and violent coughing to effect its removal; and if not removed in this state, it assumes a different one, and often becomes the cause of a fatal phthisis. Such, in general, is the state of the mucous secretions of the maxillary sinuses, when the result of slight inflammation from colds, &c.

The highly fetid condition of the discharge, at this stage of the disease, depends not so much upon an aggravated state of the disease in the membranes of the sinus, or upon its long retention in the cavity, as upon the fact of its having been secreted in a morbid state, and long retained *upon* and *around* the congested and firmly impacted mucus contained in the deep sinuses of the antrum, which, in some cases, is so extremely septic as to become green as grass, and to corrode silver. In this state, and almost from the incipient stage of the disease, the symptoms that accompany and characterize this disease, seldom differ materially from those described by almost every experienced writer upon the subject, except, however, that, although a discharge of fetid matter from the nose is a common occurrence, we have never heard of a case "when the *nose is blown from* the nostril of the affected side."

Having thus far said more, perhaps, than the subject, so far as connected with the views of my friend, Dr. H. would seem to require, our attention would naturally be directed to the therapeutical indications, or course of treatment for the cure of a disease so annoying, so extremely unpleasant, and at times so painful. But previous, however, to engaging in that department, it seems necessary, in order that our views may be perfectly understood and comprehended, to offer a few remarks upon the physiological structure or peculiarities of both the *frontal* and maxillary sinuses, in order to show how differently they are severally affected by disease, and especially the one under consideration. It is certain that they are both variously modified, presenting peculiarities of a singular character, such indeed as to convince any person that something *more than a superficial* knowledge of the general structure is necessary to secure a successful treatment of the various diseases to which they are liable.

We have the frontal sinuses of nearly a uniform, simple structure, occupying more than one-half of the breadth of the forehead, slightly interrupted with bony protuberances in different parts, all of which ought to be kept in view, where the application of the trephine is necessary in combatting disease of a certain character. We have others again, of a considerable depth, broad, extending partly over *both* orbits, and *distinctly separated* by a thick, bony partition into two cavities, the right and the left; again with no less than three separate divisions, each of which requiring, perhaps, *separate treatment,* in case of disease; but of these it is not our intention to offer any further comments at present. In the next place, the form and structure of the maxillary sinuses differ as much, if not more than those of the latter. We have them fully displayed, in which the cavity, though seldom, is of nearly an uniform shape; in the second place, they are divided into two distinct, separate and deep cavities; in the third place, into three and sometimes four separate fossæ, in the bottom or floor of the antrum, produced nearly in the following manner, viz. behind the dens sapientiæ of the superior jaw, a spinous process extends across from the external alveolus to the internal or palatine plate; between the dens sapientiæ and the second molar tooth, a second spinous process extends across in a similar manner, thereby forming a deep fossa, of nearly the size of half of a musket ball; between this, and over the second molar tooth, there is another, rather deeper and of a larger capacity; immediately over the first or anterior, large molar tooth, is another, of a somewhat larger capacity; anterior to this last, and over the second bicuspid, is sometimes another, proportioned in size to the bicuspid beneath, thus forming four; anterior to all of these, there is, in some heads, a deeper cavity than in others, stretching forwards and upwards, forming a deep fossa, extending nearly to the os unguis. We do not pretend to assert that such is the form of the maxillary sinuses in all cases. We mean to say, however, that we have the variety thus described, completely developed and exposed for examination, and which, together with the relative facts that will follow in the sequel, *were read* before the *Society of Dental Surgeons,* (my friend, Dr. C. A. Harris, being present,) at their meeting in Boston, July 22, 1842.

But, although the reading of the memoir preceded the one read by our friend, the doctor, he, in the exuberance of his magnanimity, "remembered to forget" that such an essay, or paper, or, indeed, such facts and views as it contained were ever read before the society; therefore, we may, of course, presume that they were not thought worthy of a passing remark. But of this no exceptions could be taken or made in such a case, because consistent and in perfect accordance with an established maxim, laid down and inculcated by Fresnoy, in his art of painting, a maxim which we have had occasion to use in another case, but which, however, loses none of its force by use, or of its truth or correctness by a repetition, and which any one may adopt if it suits his purpose; it is as follows:—

> "Suffer not two conspicuous lights to shine,
> With *rival* radiance, in the same design,
> But leave to *one alone* the power to blaze."

Having thus indulged in a short digression, we trust that no apology will be considered necessary, when all the circumstances are duly considered and understood.

We have remarked that the tissues of these cavities, being alike in both cases, are not unfrequently the seat of the most simple form of disease, which is that of inflammation; this, if neglected, sometimes terminates in some one of the consequences of that condition of the parts. We will endeavour now to explain the nature and result of inflammation in those tissues, more especially those of the frontal sinuses. It is, as in that of almost all other cases, that of increased vascularity and nervous susceptibility, the sure result of which is free and copious secretions of mucus, sometimes apparently of lymph, of muco-purulent matter, &c. The secretions thus accumulating in those cavities are, as already described, constantly undergoing changes from mild and bland to vitiated and corrosive. Of these we will endeavour to adduce examples, not collected from the records of *medical history,* but such as have fallen under *our own observation,* and to which *we have been* an *eye-witness.* Of these we shall only notice the most signal and striking.

It is now a number of years since a Mr. D——, with many others, both of ladies as well as gentlemen, attended an examination of the scholars composing a large school in a neighbouring town. It happened upon a pleasant day in the month of March; the rooms were crowded to excess, and, though the evening was cool, became overheated. When the exercises were over and the audience dismissed, they were ushered into an atmosphere materially different from the one they had just left. In their return home, Mr. D——, with several others, had to walk a distance of nearly half a mile. From the time of his leaving the heated room of the academy, and was ushered into the cold atmosphere of a March evening, he was seized with a chill that became general, and which increased in intensity as the evening advanced, until his return. He soon became sensible of an increasing indisposition, especially about the forehead, eyes and face. Before 12 o'clock at night, the pain and distress of the head increased, until medical assistance was urgently called for. The usual remedies generally furnished for violent colds were promptly administered, and used through the night, but with very little relief. The following day his entire head seemed to be involved in a suffering with the disease; his eyes, his ears, and, more particularly, his forehead over his eyes, were the seat of intense pain. In this state, notwithstanding all the remedies or means that could be devised were used without effect, he remained until the third day, when, all of a sudden, a free and very copious flow of a muco-purulent matter was discharged from both nostrils, which gave almost instant relief, though it did not entirely subside until the fourth day.

In this case, it is more than probable that the cavity of the frontal sinus was uniform in its structure, without any divisions or lateral sinuses, as is often the case, since, after the collection of matter had ceased, there was no sign of congested mucus following the discharge.

The following is the case of a lady of Fell's Point, Baltimore, who was lying very ill, and under the care of Dr. S. K. J——, who called upon me and said that he suspected a disease of the maxillary sinuses, and requested me to step into his carriage with him the next day, and examine the case.

We found the lady somewhat relieved from the distressing agony which she had suffered for some days, and that by a copious discharge of thick, purulent matter, mixed with pus of an extremely offensive smell, which was still draining off from the right maxillary sinus and nostril.

The case, as described by the lady, was occasioned, she believed, by a violent cold, which settled in and about her head, causing great and unceasing pain in and over her eyes, ears, and indeed, in every part of the head, and from which she could obtain no relief, until the obstruction of the nasal passage was removed, and a free passage from the antrum obtained. Under these circumstances, it was not considered necessary to adopt any special course of treatment, other than that of undisturbed rest in bed, warm, emollient applications to the head, and, in a particular manner, to avoid an exposure to a relapse. She recovered, and her health was again established in a few days.

These two cases were the result of simple inflammation in the mucous-tissue of those cavities, and to which they are always more or less liable under like circumstances, and that too, without any agency or influence of the teeth. Two other cases of a similar character occurred with two middle aged ladies, one living in the city of Baltimore, a Miss R. P——, the other a Miss S. H——, living in the precincts of the city. They had both suffered severely from violent colds that occasioned great pain and distress in the forehead and left cheek of Miss H——, and in the right side of the face and head of Miss P——.

These two cases, by judicious nursing, were soon relieved, and that without any sensible discharge from the nose. In a few weeks, however, they were both made sensible of a discharge from the nose, of a character essentially different from the common mucus, as it appeared upon the handkerchief, at the same time accompanied with a disagreeable, fetid smell, so much so as to excite the notice of the members of the family, when near them. This awakened the apprehensions of the ladies, and they made no delay in seeking advice. The cases were too well characterized to admit a doubt of their nature, and of the course that removed a considerable quantity of thick pus, but having necessary to be pursued to obtain relief; which was by injections been seasonably attended to, had not time to become congested and much less impacted in the bottom of the cavities.

The next occurrence of the kind was that of a Mrs. S——, which happened in the year 1815. This, like the preceding cases, was the result of a violent cold. The first paroxysm of swelling and pain of the cheek of the right side having mostly subsided, she was left in comparative ease, with the exception of occasional lancing pains through the cheek. The case, however, was suffered to remain long neglected and unattended to, until the atmosphere in which she moved became contaminated by the effluvia from her person, and more especially from her breath and from her nose. In this state, she applied to me for advice. I did not hesitate a moment in prescribing the only means to be resorted to for relief, which was to make an opening into the antrum, in order to give discharge to the offensive matter that it contained.

This was done with a trocar, through the base of the antrum, or in the alveolar ridge, there being no teeth in the way. An immediate and copious discharge of fetid pus was the result; but which, though considerable in quantity, did not lessen in a great degree the offensive smell, which led to the conclusion that the cavity was but partially relieved of its contents. Wishing to avoid prolixity, which would unavoidably grow out of a detailed description of the course of treatment in this case, we simply remark that injections of the most suitable kind were followed up with every attention and promptness for several days, bringing away occasionally hard lumps of offensive, congested matter. By this course, the antrum appeared to be freed from its putrid contents, and the fluids injected returned through the orifice and the nose apparently as clear as when discharged from the syringe. In this, however, we were deceived, for on the eleventh day from the commencement, we started a new mass of congested matter, alike fetid, with the previous lumps, and almost as green as grass. From this time to the complete restoration of the parts to a healthy state, every measure pursued was attended with success.

The peculiarities accompanying this case, together with that of another in which Dr. G—— had operated for a similar one, for a gentleman from the Eastern Shore of Maryland, and which case, after about seven or eight weeks, manifested itself again, with all the former symptoms, greatly excited my curiosity, and in fact induced me to engage in a critical investigation of the form and general structure of those cavities, and which resulted in the conclusion which I then drew, and which I have ever since inculcated, viz. that a just and correct description of these sinuses has, as yet, *never been given*, or if given, has never been published to the world, any assertions to the contrary notwithstanding.

Were we to continue and extend our remarks upon the numerous exceptionable points that present themselves in the essay before us, we should unavoidably be led into the business of book-making indeed, which, at present, is very far from our intentions, and no less so from our inclinations or wishes. Having thus far relied upon our own resources in the views that we have advanced; having instead of disturbing the calm repose of long neglected libraries, or the long forgotten and obsolete opinions and practice of authors never before acknowledged as orthodox, or, if acknowledged, never approved of; having, with a few exceptions, drawn from and relied upon our own resources and experience, supported or sustained by incontrovertible facts, we may proceed, we presume, without any fears of trespassing upon the legitimate bounds of propriety, in offering some remarks upon the remedial course or curative treatment which the several diseases already mentioned seem to require, and which are commonly recommended and practised.

Our friend, the doctor, suggests, as a course of treatment in cases of "muco-purulent secretion, and engorgement of the maxillary sinus," the following, viz. "1st, If the nasal opening *be closed*, the evacuation of the retained matter; secondly, the removal of all local and existing causes of irritation; thirdly and lastly, the restoration of the lining membrane," and refers us to Mr. T. Bell on the diseases of the teeth, page 259. But there seems to be a discrepancy of views between Mr. Bell and our friend, the doctor, the latter of whom, in the third indication of curative treatment, prescribes "the restoration of the lining membrane," whereas the former, in the third indication, directs "the removal of such *portions* of *bone* as may have become carious."*

Now we believe that we are safe in saying that it is a *rare occurrence* indeed that, in a case of muco-purulent secretion and engorgement, and simply of the maxillary sinus, it should be accompanied with "carious bones." We should say that there is but a slight analogy in the two cases. But our friend, the doctor, sets out to prescribe a course of treatment which is all well enough, so far as respects particular cases. But *why* not say under what circumstances the course should be adopted? Would he, or in fact any judicious practicien, if the cause of the disease was evidently the result of a cold, such as the three cases just related, commence an operation to give discharge to the contents of the antrum by extracting a sound and beautiful molar tooth; or, moreover, would any *prudent* surgeon, or dental surgeon, in case some of the teeth of the affected side should *appear* not altogether sound, recommend their removal or extraction, in order to obtain an opening into the affected sinus? Indeed we are sorry to say that we have occasion to suppose so, for our friend, the doctor, says, in a case of the kind, "*none* of the *teeth* that are in an *unhealthy condition* should be permitted to *remain*."†

* See Bell on the Teeth, page 259.

† See American Journal of Dental Science, vol. 3, p. 50.

"Tell it not in Gath."—If such are the opinions really entertained by an experienced dental surgeon, I would say, in the language of Lavater, respecting a person who hates music, the cry of a child, &c. "keep him at least *three paces distant*" from *me* in *any* event.

After having offered the remarks upon the two sentences which we have noted, the doctor starts off in search of authorities to establish the course of treatment practised in remote periods of time, and in a few instances also of a later period, even down to the publication of "The Anatomy, Physiology, and Diseases of the Teeth," by Mr. Thos. Bell. This is all well enough, and so far as an effort was intended to form a general history of all the views and opinions that have been advanced upon these subjects, might be considered an acquisition to the present fund of professional knowledge. But in this instance there is an apparent incoherence and want of consistency in so sudden a digression from the subject proposed, viz. the "Treatment &c." of the diseases of the maxillary sinuses. Moreover, the inexpedience, as well as inutility of an interruption so signal and unlooked for, seems to be aggravated by the introduction of opinions and practice of writers whose authority, as before mentioned, has never been, and probably, never will be adopted as a rule in professional practice.

For example—Amongst the authors named, the number of which extends, we believe, to thirty-seven, the practice of Weinhold is introduced, and which by reason of its extraordinary nature we will transcribe, and offer upon it a few remarks. "The method attributed to Weinhold consists in *penetrating* the sinus from the *upper* and *external* part of the *canine fossa*, with the instrument directed *obliquely downwards and outwards*, so as to avoid the branches of the infra-orbital nerves." Now our friend, the doctor, sets out, doubtless, with the intent to describe the "*treatment*," or, in other words, "the *curative indications* of muco-purulent secretions of the maxillary sinus," &c. But whether the course pursued by Weinhold was with a view to the *same disease*, or some other, is not known, certain it is, however, that should the most consummate tyro that ever attempted an operation, recommend a like course for a like disease, in the same part, we should consider him really destitute of common sense.

Let us, for a moment, look at and examine the subject. The operation proposed, "consists in penetrating the sinus from the *upper* and *external* part of the *canine fossa*, with the instrument directed *obliquely downwards*," &c. In this case it must be recollected that the canine tooth, consequently its fossa, is *anterior* and distant from *any portion* of the *antrum*, of course the last point to which any person of common sense would think of applying a trocar or a syringe for the purpose of injecting the sinus, and if he did, in a case where the *nasal passage was closed*, in what possible way would the injection be evacuated, and more especially so the muco-purulent matter contained therein? In no way that we can think of except that of hanging the patient up by the heels with the head down, that the secretions and injections might thus drain off through the downward and outward opening made by the trocar. Strange indeed—"It beats Pinnetti's conjuring all to pieces." (Pindar's story of George the Third and the dumpling.)

Such, with some others of a similar character are introduced, and as far as we can discern, for no other conceivable purpose than to swell the list of references to *foreign authorities*, while but little is said—a mere whisper as it were—of the views and opinions that have been offered upon the same subjects by our own native citizens—as a Thackston, a Hullihen, and others, whose views are inserted upon the pages of the Journal of Dental Science.

Upon the views and opinions of the various remaining authorities to which our friend, the doctor, refers, we shall wave any further remarks, in order that we may have an opportunity of expressing our own of one whose merits, and whose professional standing, as not merely a "French dentist," as my friend, the doctor, styles him, but a dental surgeon, in the true sense of the term, as far exceeds the weight of either of the authorities referred to, as the most experienced of our profession, at the present day, exceeds a common tyro—we mean M. Jourdain, of Paris.

We shall proceed to notice some of the remarks contained in the essay before us, and also the conclusions to which our friend, the doctor, arrives at, and in reply offer a few comments of our own. "Jourdain, an eminent dentist and graduate in surgery,"

says the doctor, instead of seeking egress for matter accumulated in the maxillary sinus by any of those methods; proposed, in a memoir, which he presented to the Academy, in 1765, to probe the cavity by its natural opening, and then, by suitable injections, to restore it to health.

"The subject received the attention of the Academy," and what was the result? The following, viz. "The practicability of obtaining entrance into the sinus in this way was called in question; it was contended that the difficulties presented by the peculiar structure of the parts were such that they could *seldom be overcome;* but to remove all doubt upon the subject a trial was determined on.

About this time a claim to priority of discovery of this method of treatment was set up by M. Allouel, jun. As a particular account of all these transactions are mentioned by Jourdain in his own works, and even if not so, we consider them of but little interest, and therefore, for the sake of brevity pass them by—and also the description given by Bordenave of the several instruments made use of by Jourdain, in his operations upon the maxillary sinus. Again—We have some of the doctor's own opinions, viz. "The treatment of affections of the maxillary sinus by injection through the nasal opening, having been *almost entirely abandoned*, a more *minute description of the instruments employed for the purpose is not deemed necessary.** We regret that the doctor should inculcate such an opinion, even if he believes so, for we entertain very different views on the subject, and which will be made to appear in the sequel. Moreover, the doctor informs us that in order to settle the claims of Jourdain to any merit in the case, *commissioners* were *appointed to investigate the subject,* "and who, after having made a *number of trials, came to the conclusion* that the introduction of a sound by the *nasal opening,* although, *perhaps possible*, was so *exceedingly difficult,* that it could *seldom be effected.*" (Reader, note these remarks and compare them with what is to follow.) "They attempted it," says the doctor, "upon each antrum of five subjects, and the *result proved* that the sound *pierced the membrane between the turbinated bones, more frequently* than it entered the sinus by

* Journal of Dental Science, vol. iii. p. 53.

the natural opening." Moreover, in order to add absurdity to the parent stock, the doctor says, that "Bordenave, in remarking upon this method of gaining access to the cavity, states that while the membrane between the ethmoidal and inferior turbinated bones *may* be *pierced without* causing serious injury, it induces us when it happens, to suppose that we have entered the sinus by the natural opening, which "goes to prove that the operation is as *difficult* as it is *uncertain*.* Here we are at a loss to determine which appears most deserving of *severe* animadversion, viz. the parts characterized as quotations from Bordenave, or those that are represented by the doctor as the opinions of Bordenave. It seems to afford a subject of regret that the names of the *skilful commissioners* should not have been given, to be registered on the same pages with the doctor's. Why, for the moment, let us look at this subject, for it is not without some interest as well as use. It is reported that those commissioners, in their experiments upon *five antrums*, found that the "*sound pierced the membranes between the turbinated bones more frequently* than it entered the sinus by the natural opening." Now pray what membrane was this that could be so easily pierced? Surely not the covering of the turbinated bones, which although thin, are not so easily penetrated by a sound. Then what other membrane is there that is situated *between the turbinated bones*, so as to prevent the passage of the sound or syringe. Why, perhaps it is a nondescript—a new discovery—a companion for the "ligamentum dentis." Surely, if a person was about to perform an operation for an obstruction of the lachrymal sac, by an injection, he would most probably, if *he was acquainted* with the *structure of the parts*, endeavour to find the orifice of the duct before entering the injection-pipe, and not thrust the pipe and the injection in upon the coats of the eye.

But although the subject, as represented, savours somewhat of the ludicrous, we are not disposed to indulge in a spirit of levity, but to treat it with all the gravity which it may deserve, and therefore we shall endeavour to show that the difficulty in performing the operation, according to Jourdain, is as free from danger of perforating any membrane, and as free from difficulty as that of the introduction of a catheter in *any* required case.

* Journal of Dental Science, p. 53.

That commissioners should have made such a report, we are not at all surprised at, for it would by no means be the first attempt to deprive the real discoverer of the credit of an important improvement in any branch of medicine or science; and more especially in a case like the present, because a favourable report, on the part of a dentist as the author of an improvement, would not be likely to add much to the celebrity and sagacity of the *commissioners themselves*, as members of the higher branches of the medical sciences, and sustaining a much higher stand in the *estimation* of the *public.* In corroboration of this view, look at the efforts that were made to deprive a Jenner of his well merited honours and justly acquired fame; look at the opposition that was set up and persisted in with unrelenting hostility against the discovery of the immortal Hervey, and others have experienced from those who had not given *themselves* the *trouble* to *examine* and become acquainted with the common operations in the several parts of the human body, or, if they had, they were not endowed with the capacity of understanding and comprehending the *physical phenomena common* to each and every part. But although the commissioners were appointed to examine the claims of Jourdain to any merit in this case, nay more, suppose that many may be disposed to join issue with the commissioners, as Bordenave and even our friend, the doctor, himself, in disapproving Jourdain's mode of treatment in the diseases of the maxillary sinuses, will these reports, or these opinions be sufficient to invalidate the *importance* of the *method* of Jourdain, and totally to explode the practice, and thereby deprive mankind of the essential benefits which *have been* derived from its practice? Why—it would require quadruple the number of *authorities* which *have been* and *may be named*, to accomplish the end. Let us indulge a moment in examining the effect which the practice of Jourdain had with the elite of the medical and surgical practice in Paris, amongst whom may be enumerated the most eminent medical practiciens, who have been long in the habit of calling upon Jourdain, in all cases of consultation upon the diseases of the face and head. Amongst these, the name of *Petite* stands conspicuous, as cherishing an entire confidence in the views and practice of M. Jourdain.

We come now to offer some remarks more particularly upon the several operations commonly practised for the diseases of the maxillary sinus. The several diseases to which these cavities are liable having already been mentioned, we deem it unnecessary to repeat the subject, except so far as to say that the origin, nature and variety of the causes incident to these cavities do not only require but demand different modes of treatment; as, for example, when the existence of a tumour is ascertained, whether of an indolent, fungoid or humoral, bony or fibrous character, we are well aware that injections by the nasal passage, agreeable to the method of Jourdain, would be of no real utility. In such cases the operator would see the necessity of being regulated by circumstances; some might require simply the trocar, large or small, upon the ridge or spine (where there are no teeth) of the alveolar range; others, the trephine, syringe, &c. upon different parts of the external or internal walls of the sinus, &c. But of these it is not our intention to animadvert; our business is principally with the opinions and conclusions of the very *learned commissioners* who were appointed to examine and decide upon the merits of Jourdain's method of treating certain diseases of the antrum, together with those of Bordenave and *his* proselytes.

We here see and feel the necessity of reiterating some of our previous remarks, in order that our views of the subject may be perfectly understood and comprehended. The diseases then to which we especially allude, are such as are the result, at first, of simple inflammation in the mucous and other tissues of the maxillary sinuses. The consequences of this inflammation, whether from violence or contusions, colds, dental irritation, or inflammation in the cavities, occasioned consecutively by periosteal disease in the surrounding tissues, are the *same* in *all respects*, and, when previously in a normal state, differ in no essential degree from inflammation in any other *simple mucous tissue in any part*. Under such circumstances, what is the nature and character of the secretions? At first a thin, somewhat viscid fluid, but by degrees becoming more and more viscid, and at all times susceptible, as in the nasal passages, the trachea and the bronchial cells, of *congestion*, which, under different circumstances, become tenacious, adhesive, morbid and in many cases extremely offensive, more especially when, accompanying the inflammation, ulceration of the mucous glands prevail; in this case, pus is discharged and is mixed with the congested mucus, or discharged outwardly by the nose. These secretions, as we have already observed, naturally, by their own gravity, settle down upon and into the several sinuses already described in the bottom of the antrum. The secretions so deposited, not being disturbed, become gradually impacted, more and more morbid and offensive, and, by the operations of animal heat, capable of assuming various characters, sometimes of a malignant type.

Admitting these facts as stated, what course of treatment would be suggested as most likely to obviate the difficulties and remove this gradually increasing source of pestilence? Why, the answer is ready coined; without referring it to the Sarbonne. The knight of the trocar (Bordenave) is required to perform the office of perforating the walls of the antrum in some direction to afford a discharge of the contained pus and other morbid accumulations of the sinus, and that by throwing up injections into the cavity. And what is the operation? Why, the injecting pipe is introduced into the opening made by the trocar, or, peradventure, by an opening at the extremity of some one root of a diseased tooth. And what is the result? The fluid injected is thrown, *not* upon the congested matter impacted in the bottom of the antrum, but *up against* the infra-orbiter plate, whence it is thrown *back* upon the contents of the antrum. This was the course pursued in the case of Mrs. S——, and that without disturbing or breaking up the congested matter entirely, until the *eleventh* day, as already described. It is by this reason, or this course of treatment, that the disease so frequently recurs a second and sometimes a third time. See American Journal of Dental Science, vol. 3, p. 64; and others may be referred to, if necessary. The one already related, as having been operated upon by Dr. G——, see p. 263. Such is almost invariably the result of injections thrown into the antrum from below, through an opening made by a trocar, and upon an accumulation of deposites of long standing, which are at all times the most difficult to overcome; of this *we have had* ample experience.

We will, in the next place, indulge in a few remarks upon the method first introduced and afterwards succesfully practised for many years by M. Jourdain, of Paris, but which was afterwards disapproved of by the *learned commissioners* appointed, as already mentioned, to examine the matter, and others who have adopted the same livery.

In the present instance it seems necessary to obviate the difficulty which, it is said exists, in introducing the sound or syringe by the natural nasal passage into the antrum. We would not pretend to say that a person who had *no* knowledge of the structure of the parts, or one who had never examined or knew anything of the relative situation of the natural passage and the turbinated bones, would be likely to pass either the sound or canula of an injecting instrument into the antrum with facility. But we presume to assert that a person with but little experience will meet with as little if not less difficulty in the performance of the operation, as in the introduction of an injection-pipe into the lachrymal or Eustachian tube. If then the alleged difficulties are so easily overcome, what peculiar advantages does the method of Jourdain afford, superior or equal to the common method? We reply that, in the first place, if the disease in the sinus exists, and there is no sign or indication of disease in one of the teeth of the same side, we avoid the disagreeable necessity of sacrificing a sound and valuable tooth, or more, to no earthly use or benefit; secondly, we avoid the disagreeable inconvenience of affording and establishing an exit or drain for the highly offensive matter of the antrum *through the mouth*, for the opening made by the trocar *must* be kept open and free; thirdly, we avoid all risks growing out of the admission of extraneous matter into the antrum through the artificial opening; fourthly, we are enabled to ascertain, to a certainty, the nature at least, if not the extent of the disease, whether of dropsy, muco-purulent deposites, or tumours of any kind, or abscess, without removing by the trephine, mallet and chisel, or Hays' saw, a small or large portion of the maxilla or alveolar process; fifthly, we avoid the unfavourable effects resulting through a want of due care and attention, from the admission of atmospheric air into and upon the delicate tissues of the already inflamed surface, which is excessively painful to the touch. These alone, we should suppose, are sufficient to secure an entire disapprobation of this method, if not an actual resolution or vote for expunging, with black lines drawn through the entire words.

Now for the advantages to be derived from the natural method, or from the method of Jourdain. In the first place, in a simple inflammation of the lining membrane of the antrum, a suitable injection of a detergent and somewhat anodyne quality would be all that the case would require; secondly, should it prove otherwise, however, it is an easy matter to alter and modify the nature and kind of injections as occasion may call for, as in case of *ulceration* of the mucous tissue, in which pus is most commonly discharged; thirdly, if it is ascertained that the mucous deposites established in the bottom of the antrum are firmly impacted in the several sinuses, and very fetid, the injections should be of a character calculated to arouse the energies, and restore, as far as possible, the normal condition of the tissues, at the same time, thrown in with such a degree of force as to loosen or break up the congested mucus in the bottom of the antrum; and this is the more easily and expeditiously done by this method, as the injecting pipe, when introduced into the cavity, if of a suitable curve, will throw the injection not *up through* an orifice in the *bottom* of the antrum, but *directly down* or *laterally* upon the congested mass, thus by loosening and breaking it up in pieces that, when reduced, it will pass out with the fluid injected through the nasal passage. This result is not nor *cannot* be produced by the other method; of this, *we* have had an experience sufficient to convince the most sceptical.

As our remarks have already been extended far beyond our expectations, and as we feel adverse to the introduction and multiplication of real or fictitious cases in support of our views, we shall content ourselves with but one solitary instance, which we trust will be sufficient to show not only the advantages of Jourdain's method or mode of practice, but to remove, dissipate, or do away, if possible, the flimsy vagaries founded upon the supposed difficulty of introducing the sound or injecting pipe into the maxillary sinus, whenever medical assistance may be reqired in disease of a similar character.

In the autumn of 1821, I was requested to wait upon Mrs.

Y——, who, I was informed, had for a long time suffered much from a disease of her face. On seeing her, she observed that for many months she had experienced much inconvenience from a dull pain in her right cheek, and a discharge of a fetid pus from her right nostril; that she had consulted Dr. P——, in her case, and who advised her to have a tooth extracted, for the purpose of affording a free discharge of the matter through an opening into the cavity of the cheek.

The tooth was extracted, and an opening made with a trocar into the antrum. Injections were thrown into the cavity and some pus discharged with the injection. But little relief was afforded, neither could much be expected by reason of the manner in which the operation had been performed. For example—the opening into the sinus was made by the removal of the anterior bicuspid, and afterwards by the trocar, opening a passage through the socket of the tooth. The distance from the extremity of this tooth into the sinus is so great, that it is impossible to vary the pipe of the syringe so as to throw the injection in any other direction than directly upwards into the sinus, the consequence was that all the posterior and deepest part of the sinus was beyond the reach of the injection.

As a desire was manifested that I should undertake its treatment, I consented, not however, without apprising Dr. P. of the wish of the family. Having received his approbation, I consented to engage in the treatment of the case. From the appearance of the lady, it was evident that she was far from the enjoyment of vigorous health, on the contrary of a delicate temperament, past the critical period of life—married, but had no children. In the first place, I suffered the opening occasioned by the removal of the bicuspid, &c. to heal up—and commenced by throwing mild injections of simple warm milk and water into the cavity by the nasal passage, until its contents were removed, and the injections returned through the same passage in the same state as when thrown in. I then made use of the following injection, viz. an infusion of the concrete shining soot from the back of the fireplace, nicely filtered through flannel. From the use of this article, besides deriving much advantage in restoring the healthy condition of the lining membrane of the cavity, I was in a particular manner, struck with the effects of the infusion upon the mucous glands of the lining membrane.

Believing that the sinus was restored, or as nearly so to its normal state, as there was any reason to expect under existing circumstances, I was about to relinquish all further treatment, when in one of my visits she informed me that she occasionally experienced a sharp pain, shooting from the nose through the antrum. Although no manifest indications of any hidden ailment could be discovered, I ventured another effort, knowing that in the anterior portion of the antrum, there is, as I have already described, in some cases, a deep fossa, stretching forwards and upwards by the side of the nose, into which it was not easy to throw an injection by any method. I made use of an injection better calculated to detect a diseased part than before. When I was prepared for further operation, her head being thrown back, I requested her, as soon as I had thrown in the injection, to close her nose firmly, with her thumb and finger, and, immediately to throw her head forward and downward as *low* as she could. The moment the injection passed into the fossa beside the nose, she experienced a *pungent* pain, which left not the smallest doubt that it, as was subsequently proved, was the seat of a chronic ulcer of long standing. In this state of things and at this time, I was called away to the District of Columbia, where I was likely to be detained for several weeks. Not more than a week, however, after my absence, the lady was again threatened with her former troubles. She accordingly called in a skilful physician of her acquaintance, a Dr. W——, who, by her description commenced the same treatment in the same way, except that he injected an infusion of cort. peruv. with a little laudanum, from which she obtained temporary relief. Her physician, however, Dr. W. being of a consumptive habit, was taken ill, and her assistance from that source being suspended, she was left seemingly without the prospect of relief. In this state, she had recourse to her own ingenuity and firmness, and recollecting that whenever I introduced the curved canula of the syringe into the cleft between the turbinated bones in her nose, it was always at a certain angle from the nose, she procured a suitable syringe, and while sitting before a mirror, she made the attempt, and to her own gratification, as well as relief, she succeeded in injecting the sinus, and at length became so familiar with the operation, that she has had no occasion to call in a physician, and much less either one or other of the commissioners appointed to examine into the merits of this method, or any one of their proselytes, although, as I am credibly informed, she is still living, and under the necessity, occasionally, of resorting to the same, though *"extremely difficult and uncertain,"* means for relief. So much for the opinions of commissioners, and those who without a personal examination, choose to pin their faith upon the ipse dixit, or in common parlance, the sleeve of some popular character, or high sounding name.

We regret that a want of space in the present No. of the Journal will prevent a continuance of our comments upon the several diseases enumerated by Dr. H. We hope, however, in a future No. to resume them, and to treat the several subjects presented to our notice, with that degree of candour and attention which they severally deserve; amongst which are the following, viz. caries, necrosis, softening of the bony parietes, exostosis, mollities ossium, &c. &c.

Nevertheless, limited as we are, we cannot but feel some apprehension of a mis-statement of facts in the opinions of Professor Pattison, as quoted by Dr. H. in the American Journal of Dental Science, p. 175. If we are not mistaken, the case alluded to happened in 1820, and the operation proposed was not "for the suppression of fungous tumours of the maxillary sinus, by tying the carotid artery"—but for an aneurism of the superior maxillary artery, situated behind the tuber of the left maxillary sinus. Of this case and the history of the operation, and the results, we have some *share* of knowledge. That we may be in error, we admit, but not intentionally.

