# Errata

**Published:** 1843-12

**Authors:** 


*Errata.*—In Comments upon Dr. Harris' Essay, p. 256, vol. 4, first paragraph, third and fourth lines should be transposed.

